# Observations on Various Forms of Croup

**Published:** 1826-01

**Authors:** W. Pretty


### Art. II.—*Observations on various Forms of Croup.*

By W. Pretty, Esq.

After reading the history of three cases of Croup, in your Number for October, by Dr. Gregory, which apparently indicated contagious properties, I was led to reflect upon my limited experience in that disease; and I cannot call to my recollection an instance of idiopathic or primary croup, in which I suspected the existence of such properties. I have often (for it is no uncommon occurrence) attended one child, in a family of three, four, or six children, where no restraint upon their intercourse has ever been considered necessary, beyond what is common in general attacks of illness: the same room and the same bed, in many instances, has been occupied by the unhealthy and the healthy children, without the occurrence of disease in any but the one for whom my assistance was desired.

I have at present under my care two young ladies, sisters, just recovering from croup: in both it had been brought on by imprudent exposure to cold. The elder, twelve years old, was sent home from school at Hampstead, under the impression that she was ill with hooping-cough; but I found her labouring under a violent and alarming attack of this complaint, which had existed for three days: it was soon relieved by leeches, venesection, a blister, and calomel, with compound powder of ipecacuanha. The younger was taken ill three weeks after her sister, and was immediately sent home: her symptoms were mild, and I found it unnecessary to resort to bleeding. An emetic and a purgative, followed by repeated doses of calomel and compound powder of ipecacuanha, soon removed the disease. A third sister, and who had always slept with the younger of the two that were taken ill, remained at school unaffected.

I have seen so many solitary cases of croup in families, that I cannot, at present, bring my mind to believe that it is contagious, in that form usually met with in practice, and known by the name of Cynanche trachealis. But I have witnessed many severe and fatal cases of croup, after, or rather in conjunction with, simple scarlet fever and malignant sore-throat: and here I have no hesitation in subscribing to the belief of its being contagious, but not as the primary disease. It usually came on after several days' continuance of one or other of the diseases just mentioned, and seemed to owe its production to the extension of inflammation from the fauces to the larynx and trachea in young children; and they were, to the best of my recollection, exclusively the subjects of its violence: it proved generally fatal.

Whilst practising in Kent, about nine years ago, I had an opportunity of seeing a great number of persons ill with an epidemic visitation of scarlet fever and malignant sore-throat. The young and the middle-aged, in the lower classes of society, were very generally affected by it: it was far less prevalent among persons whose circumstances in life gave them the means of guarding against its contagion. The croupy symptoms were confined to young children, adults being free from them; although among the latter some cases were very severe, in which, from sloughing in the throat, deglutition and respiration were performed with difficulty.

I obtained opportunities of examination after death; and in all who died with croup as a symptom, (and I recollect none that died without it,) I found extensive inflammation of the fauces, larynx, and trachea. The adventitious membrane commonly found in fatal cases of cynanche trachealis was present in many instances; in others, only a copious exudation of thick mucus; and in some, a positive sloughing of the tonsils, velum palati, and epiglottis. I well remember one dissection of a child, about five years old, where I found the last-described state of parts, with the addition of ulceration in the interior of the larynx, and a perfect lining of the tracheal tube a considerable way down towards the lungs, formed of coagulable lymph. I might here observe, that the scarlet efflorescence on the skin was wanting in some cases of the disease in adults; and that those who had it not, or but faintly so, generally had the worst throats. The same contagion produced in some the scarlet fever, in others the malignant sore-throat.

In town practice, I have met with two well-marked instances of the absence of the scarlet efflorescence, when every other symptom of illness corresponded with the cases of scarlet fever which I had then under treatment in the same house and family. Here there was no aggravation of inflammation in the throat, and the recoveries were quick and satisfactory. I have never seen croup follow scarlet fever in London; nor do I recollect ever attending a case which proved fatal, where timely assistance was obtained.

I feel disposed to think that common and slightly ulcerated sore-throats are sometimes contagious. I was summoned, early one morning, to a child in a respectable family, who had been ill of sore-throat for several days, or a week: he had not received medical assistance, in consequence of his great aversion to taking medicine. I found him in a sinking state, from a frightful sloughing of the fauces, and he died in the evening of the same day. Subsequently four other children, brothers and sisters of the deceased, were attacked with cynanche tonsillaris, but without one unfavourable symptom, and they soon recovered. I have seen many such instances of apparently contagious properties in this disease.

From the evidence I have adduced in my own experience of croup following scarlet fever and malignant sore-throat, I should think that the cases recorded by Dr. Gregory must have been of a similar nature, particularly as he says that the first child he found very ill with "ulcerated sore-throat." Under such circumstances, I believe him to have been quite correct in supposing the disease he was attending to be contagious.

Perhaps your readers will pardon me if I extend this communication by offering a few observations upon another species of croup, to which young children are subject, and concerning which medical men are a little divided in their opinion. I allude to that peculiar species of convulsive disease named Cerebral Croup, and which is described by the late Dr. Clark, in his "Commentaries on the Diseases of Children." I was first engaged in endeavouring to understand this complaint, in consequence of two of my own children becoming the subjects of it. One of them, after having been for several weeks affected, at indefinite times, with paroxysms of spasms, accompanied with a croupy inspiration, but apparently otherwise in the possession of health, with the exception of an occasional bowel-complaint, was seized with two slight convulsions in the morning; a third occurred in the afternoon of the same day, and which terminated her existence almost instantly. Upon dissection, the blood-vessels of the brain were found unusually loaded, and effusion had taken place into the ventricles, and between the arachnoid and pia mater. Four introsusceptions of the ilium, without any evidence of local inflammation, were also discovered; but no other appearance of disease. My second child was seized with a convulsion a week after the death of her sister, (they were twins,) which was followed by repeated paroxysms of croupy and impeded respiration, which threatened the production of another general convulsion; symptoms of meningitis supervened, as also those of effusion,—such as the peculiar motion of the arm about the head, frequent startings with screamings, insensibility, squinting and dilated pupil, &c. These very formidable symptoms were removed by leeches to the temples, blistering plasters to the neck and head, with calomel purgatives; but the croupy paroxysms continued for several months, when they ultimately yielded to the exhibition of mistura assafœtida, which was prescribed by a medical friend. It appeared here to have been continued by the force of habit, the original cause having been removed long before the spasmodic actions were overcome. The age of these children, when attacked with convulsions, was eight months.

My third child, when seven months old, was taken ill like his sisters. The nurse who had the care of him neglected to give him proper exercise, and moreover frequently fed him with spoon-food, when he ought to have had the breast. He was occasionally disordered in his bowels, as most children are during infancy; and, for some time prior to his illness, he could never bear that active exercise in the arms which a good nurse will always give to a healthy child, without the respiration and circulation becoming so seriously impeded as to give alarm for his safety to all present. The attack of croup was preceded by a few days of febrile excitement, with cough, and sudden startings without any apparent cause. The treatment consisted of the application of leeches, with the exhibition of aperient and saline medicines. He improved under this plan, but the spasms did not leave him. A few weeks after this illness, he was seized with a convulsion, and the same means were again resorted to, with equal benefit; but, the spasms frequently recurring, I was constantly in fear of a repetition of convulsion. I had the child weaned, and sent him a few miles out of town; where, under the care of a more trust-worthy nurse, he gradually lost the complaint, and is now a fine healthy little fellow.

Not fully understanding the nature of this affection, I took every opportunity that presented of asking my medical friends for their opinion; and I am sorry to say that I was, if possible, more perplexed than before, for I scarcely found any two of them to agree, though several stand deservedly high in their profession, and I much respect them for the possession of superior medical talents. I trust that, without giving offence to any, I may say, that by one it was considered to arise from the cutting of a tooth, the obvious remedy for which was lancing the gums: this was done, but no tooth was cut till two months after the complaint had disappeared. The wet-nurse was also recommended to be changed. By another it was a cerebral affection, and serious in its consequences. A third gave it as his opinion that gastric and intestinal irritation was the cause, without any particular reference to the head. By a fourth it was supposed to depend upon local pressure on the recurrent nerve, or its branches: an external application was recommended, and some alterative medicine prescribed, and the child to be weaned.

About this time the disease had accidentally excited a good deal of attention, and some excellent communications appeared in the different Medical Journals; but concerning its pathology and treatment there seems still to exist a great discrepancy of opinion. Upwards of a dozen cases have been under my care, independent of the experience I have had under my own roof; and in all I have seen such powerful reasons for believing the affection to be produced by cerebral irritation, that I do not hesitate to give it as my opinion that, in by far the majority of cases, the encephalon was the seat of the complaint; and, although the cutting of a tooth, or the irritation arising from disordered bowels, may occasionally prove the exciting cause, that it mainly depends upon something wrong within the head. In confirmation of my opinion, I would draw the attention of your readers to a paper upon Meningitis, in the "Medical Repository," written a few months since by Mr. DAVIES, in which the symptoms there detailed as characteristic of that disease have been more or less present in all acute cases of this species of croup that have come under my observation.

These spasmodic attacks are very generally the precursors of convulsions, unless means are adopted to prevent them, and then children die of meningitis. This fatal course happened to a child in a family where another had previously died of meningitis, and subsequent effusion. A third child in this family had the complaint a year after, and it continued for three or four months, during which time he experienced two seizures of convulsions, and the latter have more than once returned along with the croupy inspiration, when his system has appeared surcharged with blood ; and this was very likely to happen, from being too much indulged with food. Bleeding, purgatives, and blisters, with cold lotions to the head, were the means chiefly used for his relief. The spasms continued for several months, gradually losing their violence and frequency; and he is now five years old, and a fine healthy-looking boy. He struggled through a smart attack of continued fever this last spring, which affected his head severely, but produced no return of his former symptoms.

I have also to add, that I lost, in April last, an infant, only three months old, in the same family, which was affected with croupy respiration, repeated convulsions, and every symptom of meningeal inflammation. This infant's alvine discharges had a very unhealthy appearance during its illness, and I have frequently had reason to suspect that such depraved secretions were continual as effects of such seizures. The eldest child, now ten years old, has experienced two attacks of epileptic convulsions within the last two years, and each time was cured chiefly by depletory measures. The parents of this family have indeed been unfortunate with their children: but such afflicting results are not always met with, as I have experienced the pleasure of seeing recovery effected by the same means in more than half the cases that have come under my care.

Reflecting, as I have often done, upon these and similar cases, I cannot avoid indulging a certain degree of belief that their production is very often intimately connected with hereditary conformation: in proof of which opinion I can say, that the mother of the family just instanced as suffering so much from this complaint, is, without exception, one of the most timid and nervous females I ever knew, and is almost daily complaining of headache.

I will also mention, that the father of a child which has recovered from two attacks with convulsions, and of another which died suddenly in a convulsion, always exhibited a certain weakness of his mental powers, and is now unhappily bereft of his reason: he is in a state of childishness. More evidence of the same kind I could adduce, but, to my mind, the fact is quite obvious.

The object I have had in view in relating these cases, is to show that, however necessary it may be to attend to the state of the bowels in this disease, as well as the state of the gums during the period of dentition, it is much more so to attend to that of the brain and nervous system, which will generally require the loss of blood before recovery can be effected.

I will close this communication by an answer to an observation I have heard, that young children cannot lose blood with impunity. Now, this must very much depend upon the extent to which the evacuation is carried. It has twice occurred to me to consider it necessary to recommend the application of leeches to infants within forty hours after their birth: one in consequence of pressure sustained during parturition, producing convulsions, which would not yield to every other means that could be thought of; and a second time where the same cause, in a breech presentation, produced a state of apparent asphyxia, which artificial respiration and the warm bath imperfectly removed, but which was for twenty hours followed by impaired sensibility, impeded respiration, screaming, and starting of the fingers from spasmodic action of the extensor muscles, and a disinclination to suck. Both infants were most satisfactorily relieved by the bleeding.

*Mabledon-place; Nov. 8th,* 1825.

